# Ovariotomy and Marriage

**Published:** 1888-10

**Authors:** 


THE BOSTON *Medical and Surgical Journal*, in commenting on ovariotomy and marriage, uses the following significant language: "Women may and do marry without the husband being necessarily aware of the hiatus in the anatomical arrangements of his better-half. A marriage contracted under such circumstances would from any point of view be immoral and contrary to public policy, and when one of the parties to the contract is left in ignorance of the facts of the case, it becomes a fraud of the most cruel description. Moreover, except with the assistance of the surgeon who was instrumental in removing the superfluous organs, it would be difficult for the outraged husband to prove his case, should he be tempted to resort to the law to relieve him of a mutilated and infertile conjoint. In years gone by, the question was one which might well be left to the debating societies to discuss, but in view of the extension which the practice of extirpating ovaries has taken of late years, it becomes one of practical interest. If it be hard on the man to give him a wife minus her appendages, it is adding insult to injury to give him for a wife a woman who has not even a uterus to call her own. Yet Mr. Tait was enabled to record the cases of three young women, wombless and appendageless, who had subsequently reported favorably on the matrimonial condition. Where the operation in the case of either conjoint has been performed subsequently to marriage, no difficulty occurs, since each party to the contract has solemnly consented to accept the other for better or worse, but just as lepers are only permitted to marry *inter se*, so the only fit spouse for the woman without ovaries would be the man without testicles."

